# Individual differences in rat sensitivity to CO_2_

**DOI:** 10.1371/journal.pone.0245347

**Published:** 2021-01-22

**Authors:** Lucía Améndola, Anna Ratuski, Daniel M. Weary

**Affiliations:** Animal Welfare Program, University of British Columbia, Vancouver, British Columbia, Canada; National Institute on Drug Abuse, UNITED STATES

## Abstract

Feelings of fear, anxiety, dyspnea and panic when inhaling carbon dioxide (CO_2_) are variable among humans, in part due to differences in CO_2_ sensitivity. Rat aversion to CO_2_ consistently varies between individuals; this variation in aversion may reflect CO_2_ sensitivity, but other personality traits could also account for individual differences in aversion. The aims of this study were to 1) assess the stability of individual differences in rat aversion to CO_2_, 2) determine if individual differences in sweet reward motivation are associated with variation in aversion to CO_2_, and 3) assess whether variation in aversion to CO_2_ is related to individual differences in motivation to approach gains (promotion focus) or maintain safety (prevention focus). Twelve female Sprague Dawley rats were exposed multiple times at three different ages (3, 9 and 16 months old) to CO_2_ in approach-avoidance testing to assess motivation to avoid CO_2_ against motivation to gain sweet rewards. Rats were also tested for motivation to find hidden sweet rewards, and for their motivation to approach rewards or darkness. Tolerance to CO_2_ increased with repeated exposures and was higher at older ages. Individual differences in aversion to CO_2_ were highly repeatable but unrelated to motivation for sweet rewards or the strength of promotion and prevention focus. These results indicate that individual differences in aversion to CO_2_ reflect variation in CO_2_ sensitivity.

## Introduction

People report feelings of fear, anxiety, dyspnea and panic during CO_2_ inhalation (for a review see [1]). This emotional response to CO_2_ inhalation varies among individuals. With a single inhalation of 35% CO_2_, around 24% of healthy humans experience panic attacks [2, 3]. Between 43 to 94% of panic disorder patients experience PAs after a single inhalation of 35% CO_2_ [1]. When inhaling 35% CO_2_, the anxiety experienced by healthy people and the panic attacks experienced by panic disorder patients are highly consistent between exposures [4, 5]. This between subject variability in the subjective emotional experience is often referred to as variation in CO_2_ sensitivity [1].

Rats respond to CO_2_ exposure with defence behaviours [6–9], and are motivated to avoid this agent [9–11]. Rats have also been used as translational models for understanding the underlying mechanisms of the emotional response to CO_2_ inhalation [12, 13]. Rat behavioural responses to CO_2_ are highly variable. For example, during forced (i.e. unavoidable) exposure to CO_2_ “escape attempts” have ranged between 0 to 34 among rats [7], with 50% of rats showing an increase in locomotion [9], and 20% of rats moving around the cage perimeter [14]. Aversion to CO_2_ is also variable among rats. For example, in one study the latency to avoid CO_2_ varied between individuals from 7 s to 48 s in an aversion-avoidance setting (in which the cost of avoiding the CO_2_ delivered in a dark compartment was escaping to a CO_2_-free compartment that was brightly lit) [10]. In an approach-avoidance setting (in which the cost of escaping to a CO_2_-free compartment was the loss of sweet rewards), the threshold of aversion ranged between 11 to 19% CO_2_ between rats [9, 11]. In more recent work we found that variation in rat behaviour was consistent between two exposures to CO_2_, during forced exposure, aversion- and approach-avoidance testing and we found that rats that consistently showed higher responses to CO_2_ forced exposure were consistently less tolerant of CO_2_ when tested in aversion-avoidance [15]. These results suggest that variation in rat responses to CO_2_ is linked to consistent individual differences in CO_2_ sensitivity; i.e. like humans, rats may vary in the emotional experience elicited by CO_2_ (for a review, see [16]). The first aim of the current study was to determine whether individual differences in rat aversion to CO_2_ in approach-avoidance tests assessed at 3, 9 and 16 months of age, are stable and consistent through multiple exposures.

Individual differences in aversion to CO_2_ could be caused by behavioural differences elicited by testing contingencies specific to the approach-avoidance setting. In approach-avoidance tests, exposure to CO_2_ is paired with access to sweet rewards that rats are motivated to approach [11]. An underlying but untested assumption is that the strength of motivation to approach the sweet rewards is similar among rats. However, motivation for sucrose is known to consistently vary among rats [17–19], so it is possible that variation in rat aversion to CO_2_ in approach-avoidance tests is due to individual variability in motivation for sweet rewards. Thus, the second aim of our study was to assess if individual differences in rat responses to CO_2_ in an approach-avoidance test are associated with variation in sweet reward motivation.

Following regulatory focus theory [20], variation in rat behaviour in approach-avoidance could be related to individual differences in the strength of promotion and prevention motivations. Individuals focused on promotion are more motivated to approach gains and are more sensitive to their presence or absence; individuals focused on prevention are more motivated and sensitive to safety related incentives [21]. Promotion and prevention motivations are independent; hence individuals can be high or low in promotion or prevention motivations, or both [22]. These motivational foci are consistent over time in rats [22, 23]. Approach-avoidance tests involve both gain (sweet rewards) and safety (CO_2_-free cage) incentives. Individual differences in promotion and prevention motivations could account for variation in CO_2_ thresholds of aversion. Promotion focused individuals may tolerate CO_2_ concentrations to maximize gains, and prevention focused individuals may be maintaining safety by avoiding non-threatening CO_2_ concentrations. Thus, the final aim of this study was to determine if individual differences in regulatory focus are related to variation in CO_2_ aversion in approach-avoidance.

## Methodology

All procedures were performed in accordance with the guidelines on care and use of rodents in research established by the Canadian Council on Animal Care and were approved by The University of British Columbia Animal Care Committee (protocol A15-0071).

### Subjects and housing

In a previous study by our group, individual differences in rat responses to CO_2_ were detected with a sample size of 12 rats [15]; therefore, 12 Sprague-Dawley rats were used in this study. Rats were obtained as surplus stock from the University of British Columbia as an effort to reduce the total number of animals used; however, only female rats were available at the beginning of the current study. Rats were 3 months old and weighed 328 ± 45 g (mean ± standard deviation) at the beginning of the study. All subjects were marked with an animal marker (Ketchum Manufacturing Inc., ON, Canada) and housed in groups of three on a 12 h light/dark cycle at controlled temperature and humidity (21 ± 0.4°C and 52 ± 11%, respectively). The housing system consisted of two cages (20 cm x 50 cm x 40 cm) connected by a red polycarbonate tube (7.6 cm diameter, 15.0 cm long). Both cages contained bedding (Biofresh, Absorption Corp, WA, USA) and environmental enrichment (e.g. cardboard boxes, hammocks, PVC pipes, and shredded paper towels). Rats were provided ad libitum food (Rat Diet PMI 5012, Lab Diets, Land O'Lakes, Inc., MN, USA) and tap water. Rats were also provided daily access to a playpen (i.e. a highly enriched large cage; following [24]) for 30 min/d.

#### Handling and transport

Rats were habituated to handling during the week before experiments started (following [24]). For all sessions, rats were individually transported in a cage covered with black plastic. All habituation, training and test trials were conducted during the light cycle between 900 h and 1700 h. Each rat was habituated, trained, or tested only once per day at similar hours each day across all tests, and only separated from cage-mates for a maximum of 40 min per day. Test cages and the apparatus were cleaned with a combination of water and isopropanol (70%), and bedding was replaced before the next session.

### Experiment 1: Aversion to CO_2_

#### Apparatus

**Fig 1:**
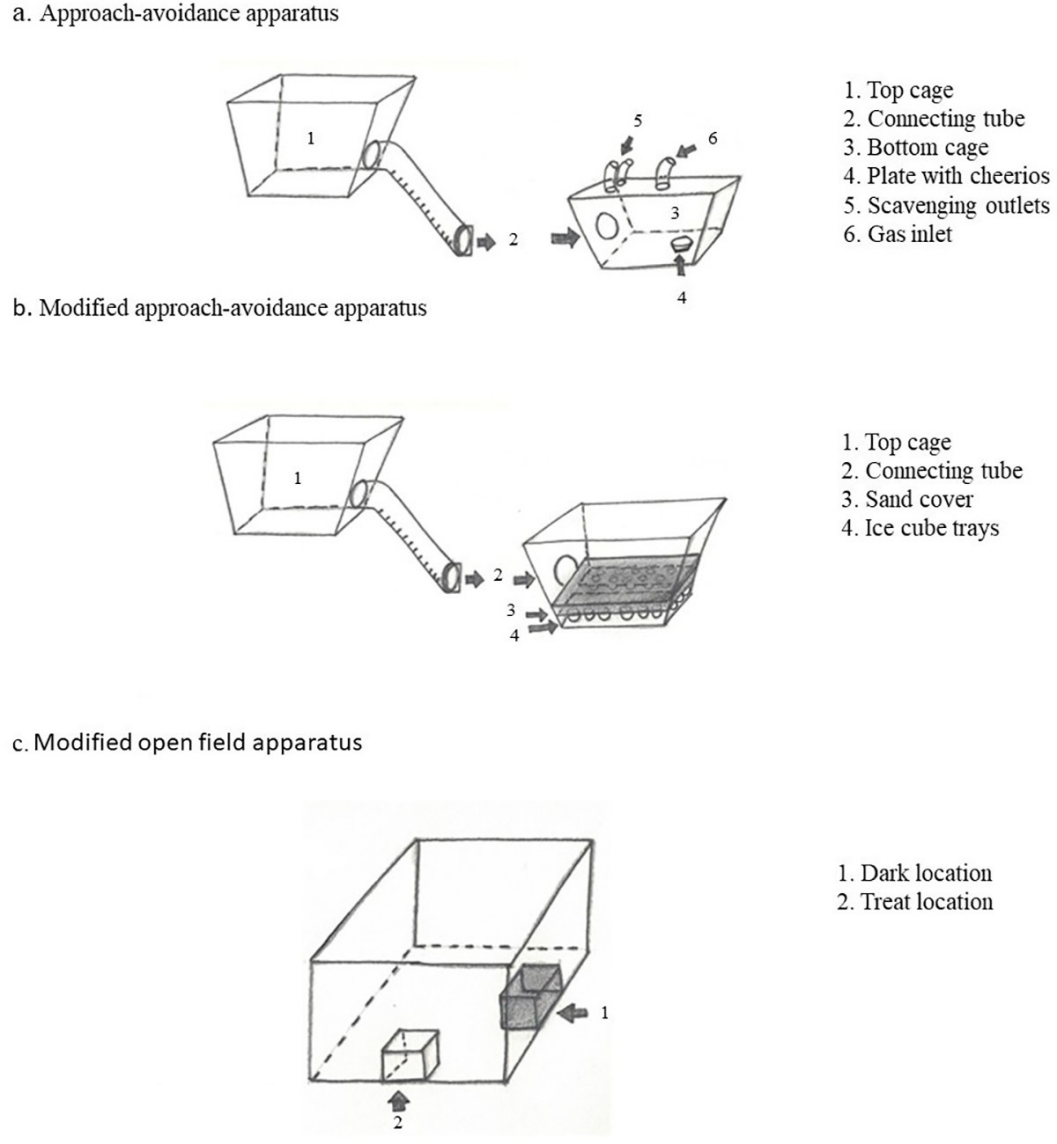
Experimental apparatus. a) Approach-avoidance apparatus used to assess aversion to CO_2_. Measurements were: The top cage 20 cm x 50 cm x 40 cm, bottom cage 20 cm x 45 cm x 24 cm, connecting tube 10 cm diameter x 45 cm long, and plastic sliding door 10 cm x 10 cm. b) Modified approach-avoidance apparatus used to evaluate motivation for sweet rewards, the test cage measured 20 cm x 45 cm x 24 cm and the ice cube trays 32 cm x 12 cm x 4 cm. c) Modified open field arena used to assess promotion and prevention motivation focus, the arena was made of white acrylic glass (100 cm x 100 cm x 61 cm) and contained two smaller acrylic glass boxes (10 cm3) placed against the center of two adjacent walls of the arena (treat and dark locations).

An approach-avoidance apparatus was used to assess repeatability in aversion to CO_2_ through multiple exposures. The apparatus consisted of a larger top cage (one of the rat home cages) positioned 20 cm above a smaller bottom cage (Fig 6A). Both cages contained bedding. The top cage was connected to the bottom cage via a transparent acrylic tube fitted with plastic cleats to improve traction. A plastic sliding door was attached to the entrance of the connecting tube at the top cage of the tube. The top cage was covered with a wire lid, and the bottom cage was covered with a clear acrylic lid. To allow for comparability between studies, gases were delivered from an inlet in the acrylic lid [9, 11, 15, 24]. The lid also contained two scavenging outlets inlet (Fig 1A); the scavenging outlets were connected to a wall exhaust system by a plastic scavenger hose.

Two flow meters (CO_2_: Western Medica, OH, USA; air: Dwyer instruments, Inc., NI, USA) were used to regulate gas flow. Gases (air and CO_2_) were delivered from compressed gas cylinders (Praxair, BC, Canada), through a clear vinyl tube inserted in the gas inlet.

#### Habituation, training and testing procedures

Subjects were trained to eat 20 sweet rewards (Cheerio; Honey Nut Cheerios TM, General Mills Inc., MN, USA) in the bottom cage of the apparatus. At the beginning of each training session, the subject was placed in the top cage and allowed to explore the apparatus for 5 min, with air (4 L min^-1^) flowing into the bottom cage at all times. The experimenter then tapped her fingers on the side of the cage, gave the rat one sweet reward in the top cage, and closed the sliding door to block access to the bottom cage. The sliding door remained closed for 60 s while 20 sweet rewards were placed in a dish in the bottom cage. The sliding door was opened and the rat was able to descend into the bottom cage to consume the sweet rewards. The training session ended as soon as the rat left the bottom cage (i.e. shoulders crossed into the tube exiting the bottom cage). A rat was considered to have met the training criterion when, during three consecutive training trials, it stayed in the bottom cage for 5 min or ate all 20 sweet rewards, whichever occurred first.

**Fig 2:**
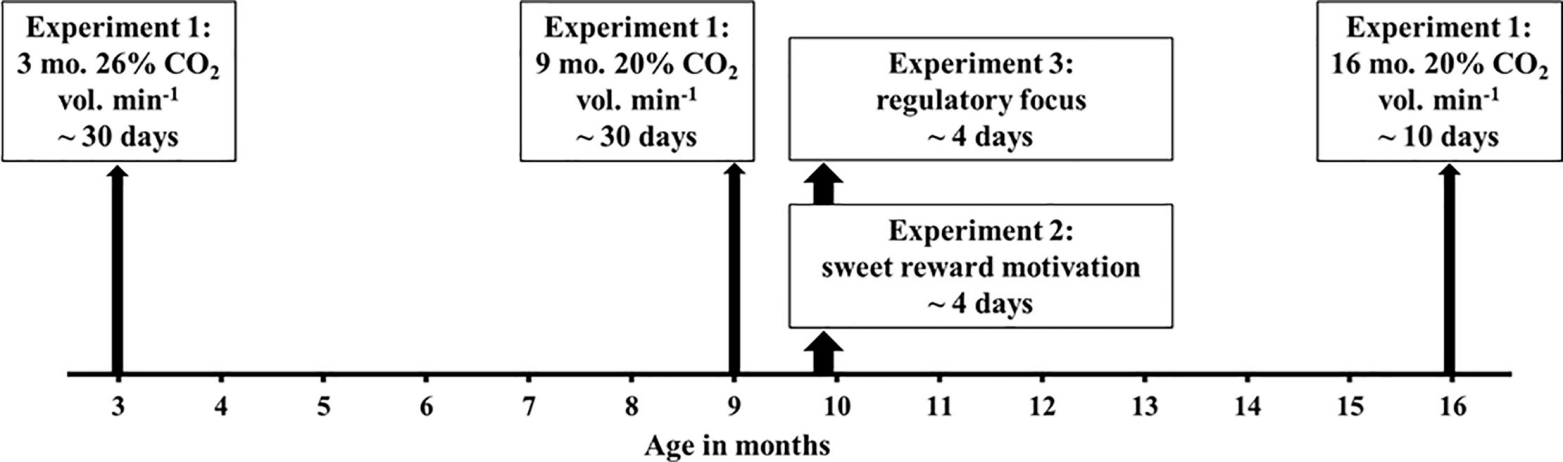
Experiments timeline. Order of testing across the three experiments (i.e. aversion to CO2, sweet reward motivation and regulatory focus). In all three experiments rats were trained, retrained or tested every weekday but not on weekends.

Once trained, rats were repeatedly exposed to CO_2_ gradual-fill (3 months of age: 26% CO_2_ cage vol. min^-1^; 9 and 16 months of age: 20% CO_2_ cage vol. min^-1^) in the approach-avoidance apparatus. During CO_2_ trials, air flow was substituted with CO_2_ flow as soon as the rat started eating the sweet rewards. We ran one control trial (air flow of 4 L min^-1^) after every two CO_2_ exposures. The trial ended when the rat left the bottom cage. Latency (s) to exit the bottom cage was recorded by direct observations. If a rat failed to stay for 5 min or eat all 20 sweet rewards in a control trial, the previous CO_2_ trial was excluded and the rat was re-trained before continuing in CO_2_ trials. Rats were re-trained until the training criterion was met at 9 and 16 months of age (for order and length of the experiments, see Fig 2).

#### Assessment of CO_2_ concentrations

With no animal present in the approach-avoidance apparatus, we conducted twelve CO_2_ flow trials for each of the two flowrates used (26% CO_2_ chamber vol. min^-1^ and 20% CO_2_ chamber vol. min^-1^) to assess changes in CO_2_ concentration during gradual-fill. A clear plastic sampling tube, connected to an oxygen analyzer (Series 200, Alpha Omega Instrument Corporation, RI, USA), was inserted through the inlet in the middle of the acrylic glass lid. The oxygen analyzer readings were video recorded during filling (5 min). Every 0.2 s CO_2_ concentrations were estimated from changes in oxygen concentrations using the formula CO_2 (t = x)_ = 100 –([O_2 (t = x)_ * 100] / O_2 (t = 0)_.

### Experiment 2: Sweet reward motivation

#### Apparatus

A modified approach-avoidance apparatus was used for this test. During baseline, the apparatus remained the same as described for the approach-avoidance test. During test sessions, the bottom cage was replaced with a new test cage measuring 20 cm x 45 cm x 24 cm. The test cage was covered with a wire lid and contained two ice cube trays with 12 holes each, and was covered with autoclaved sand (Fig 1B).

#### Training and testing procedure

Rats were habituated once and tested three times for sweet reward motivation. At the beginning of each session, the rat was placed into the top cage, and could freely move between the top and bottom cages for 5 min. The rat was then given a signal by the experimenter to receive one sweet reward in the top cage, and the sliding door was closed for 60 s. During this period the baseline bottom cage was replaced with the test cage. The test cage contained 20 sweet rewards placed in ice tray holes and hidden underneath a layer of sand. One sweet reward was left on top of the sand in the middle of the cage. The rat was then allowed to descend to the bottom test cage to search for and consume the rewards. The session ended if the rat left the test cage (i.e. shoulders crossed into the tube exiting the cage) without carrying a sweet reward, or if the subject had left the cage carrying a sweet reward but did not return to the test cage within 3 s of having consumed the sweet reward.

For the training trial, the sweet rewards were distributed in 6 adjacent holes of the ice tray, with 3 to 4 sweet rewards per hole. For rats that consumed fewer than 15 sweet rewards during their training trial (n = 5 rats), training was repeated a second time. In the first test trial, the sweet rewards were distributed into 9 reward holes, separated by empty holes, with 2 to 3 sweet rewards per hole. In the second test trial, sweet rewards were evenly distributed throughout the tray with only one sweet reward per reward hole and at least one empty hole between each reward. In the third test trial, the sweet rewards were randomly distributed throughout the tray at coordinates obtained from a random number generator with a maximum of 2 rewards per hole.

Any rewards remaining were counted at the end of the trial. All trials were video recorded and scored using Boris software (Version 7.0.9) [25]. A trained observer, blind to rat identity and trial number, scored the videos for the number of sweet rewards consumed and searching time between each consecutive reward found (s). Inter-observer reliability was estimated from 10 videos scored by the trained observer and another independent observer (number of sweet rewards consumed: r = 0.99; searching time: r = 0.99).

### Experiment 3: Regulatory focus

#### Apparatus

Following Franks and colleagues [23], a modified open field arena was used for regulatory focus profiling. The modified open field arena was made of white acrylic and contained two smaller acrylic boxes placed against the center of two adjacent walls of the arena (treat and dark locations; Fig 1C). The arena was illuminated with red light and white light that provided an average light intensity of 82 ± 1.6 lux (mean ± standard deviation) at the center of the arena floor.

#### Habituation and testing procedure

Rats were habituated twice and tested twice in the modified open field arena. Before each trial, a variety of food rewards (20 Cheerios: Honey Nut Cheerios TM, General Mills Inc., MN, USA; 10 sunflower seeds: Raw Sunflower Seeds, Western Family, Overwaitea Food Group LP, BC, Canada; 1 yogurt drop: Drops Yogurt Flavoured Treats, Living World, Hagen Inc., QC, Canada; 2 peanut M&Ms: Mars Canada Inc., ON, Canada; 20 peanuts: Peanuts Roasted in the Shell, Western Family, Overwaitea Food Group LP., BC, Canada) were placed inside of one of the small boxes (treat location) inside the arena, and the other small box was left empty (dark location). At the beginning of each trial, the subject was introduced to the arena in the farthest corner from and equidistant to the treat and dark locations. Rats were left in the arena for 10 min; if the rat approached the dark location (within one body length) the light would turn off for 30 s or until the rat left the dark location, whichever occurred first.

All trials were video recorded and scored by a trained observer, blind to rat identity and trial number. The size of rats was bigger than that of the treat and dark locations; therefore, and following Franks et al. [23], the observer scored the amount of time rats spent within 20 cm of the treat and dark locations (s) using Boris software. Another independent observer scored 4 videos to estimate inter-observer reliability (treat location time: r = 0.99; dark location time: r = 0.99).

### Data analysis

Analyses were carried out with R (R Development Core Team, Version 3.4.1) and RStudio (RStudio, Inc., Version 1.0.136). The model residuals and data were visually assessed for normality. Results are reported as mean ± standard error.

#### Experiment 1: Aversion to CO_2_

We estimated the CO_2_% concentrations at the time when rats exited the bottom cage (i.e. CO_2_% avoided), using the average concentration of CO_2_ at each time point (measured every 0.2 s) during the 12 CO_2_ flow trials. Random effects models are a useful tool to handle unbalanced or incomplete data, and it has been shown that the power to detect individual differences in these models is improved by the inclusion of data from individuals with only a few observations and that removal of individuals with a low number of observations is unjustified [26]. Hence, we used all available observations from all subjects using a linear mixed model (“nlme” R-package; an alternative analysis considering each age separately is presented as Supporting Methods S1 in S1 File). The model presented here, included the response variable CO_2_% avoided in approach-avoidance tests, age (3, 9 and 16 months of age) as a fixed factor, exposure number (within age) as a covariate, the interaction between age and exposure number, and series identity (i.e. unique combination of the individual rat by the age at which observations were taken) within rat identity as a random intercept. We found that weight as fixed effect did not significantly affect aversion to CO_2_, hence was not included in the model (results not shown). Age and exposure number were both mean centered and standardized to 2 standard deviations [27]. We assessed the fit of this model as essentially equivalent to a model that included exposure number as a random slope and series identity within rat identity as random intercept (see Supporting Methods S2 in S1 File). We assessed the power to detect significant random intercepts and slopes given the current study sampling structure [26] and found sufficient power (0.87) to detect differing random intercepts by rat but low power to detect differing slopes by rat (Supporting Methods S3 in S1 File; further model diagnostics are presented in Supporting Methods S4 in S1 File). The significance of the random intercept was evaluated using the likelihood ratio test (LRT). We estimated repeatability (R; “rptR” R-package) of CO_2_% avoided adjusted for age and within age exposure number (adjusted repeatability for Gaussian data [28]). The point estimate of aversion to CO_2_ for each individual rat was calculated as the average best linear unbiased predictors (BLUPs) of the random effects obtained from 1000 simulations (“arm” R-package). This method has been used in behavioural ecology studies to reduce biases in the estimates, arising from for example habituation or increased number of repeated tests in older animals [29].

#### Experiment 2: Sweet reward motivation

To assess individual differences in sweet reward motivation, the total number of rewards consumed, and the total searching time were included as response variables in two linear mixed models. In the models, trial number was included as fixed factor and rat identity as random intercept. LRTs were used to assess the significance of the random intercept, and repeatability (R) across trials was assessed.

We then estimated the average number of sweet rewards consumed and total searching time per rat across trials. The relationship between the two measures of rat motivation for sweet rewards and the average BLUPs of CO_2_% avoided in approach-avoidance tests was assessed using Pearson correlation tests.

#### Experiment 3: Regulatory focus

To assess consistency in promotion (and prevention) focus, we used Pearson correlation to examine the percentage of time spent in the treat (and dark) location in the two test trials. For each rat, we estimated the average percentage of test time spent in the treat (and dark) location across the two trials. Again, we used Pearson correlation to assess the relationship between promotion (and prevention) focus and the average BLUPs of CO_2_% avoided.

#### Sample disposition

For Experiment 1, some rats failed to meet training criterion after six training trials in approach-avoidance within each age; these rats were not tested. We tested nine, nine and six rats at 3, 9 and 16 months of age, respectively. At age 16 months, eight rats were re-trained in approach-avoidance; however, two rats had to be euthanized due to mammary tumor development. The remaining six rats were clinically healthy. Due to repeated failure to meet training criterion during control trials (four consecutive trials), not all rats were tested with CO_2_ for the same number of exposures at each age (see Table 1). For experiments 2 and 3 we tested 11 rats–one of the rats tested in approach-avoidance failed to follow handling procedures and was excluded from these experiments. We used these 11 rats to assess the relationship between aversion to CO_2_, sweet reward motivation and regulatory focus (see Table 1).

**Table 1:** Sample disposition for experiments 1 (aversion to CO_2_), 2 (sweet reward motivation) and 3 (regulatory focus), specifying number of exposures or trials for each individual rat and the age at which rats were tested.

	Experiment 1			Experiment 2	Experiment 3
	Number of exposures to CO_2_			Number of trials	
Rat identity	3 months	9 months	16 months	9 months	9 months
1	0	4	3	0	0
2	9	9	0	3	2
3	10	9	0	3	2
4	7	0	0	3	2
5	9	9	3	3	2
6	10	0	0	3	2
7	10	0	0	3	2
8	0	9	0	3	2
9	7	7	2	3	2
10	0	10	3	3	2
11	10	10	3	3	2
12	10	9	3	3	2

## Results

### Experiment 1: Aversion to CO_2_

**Fig 3:**
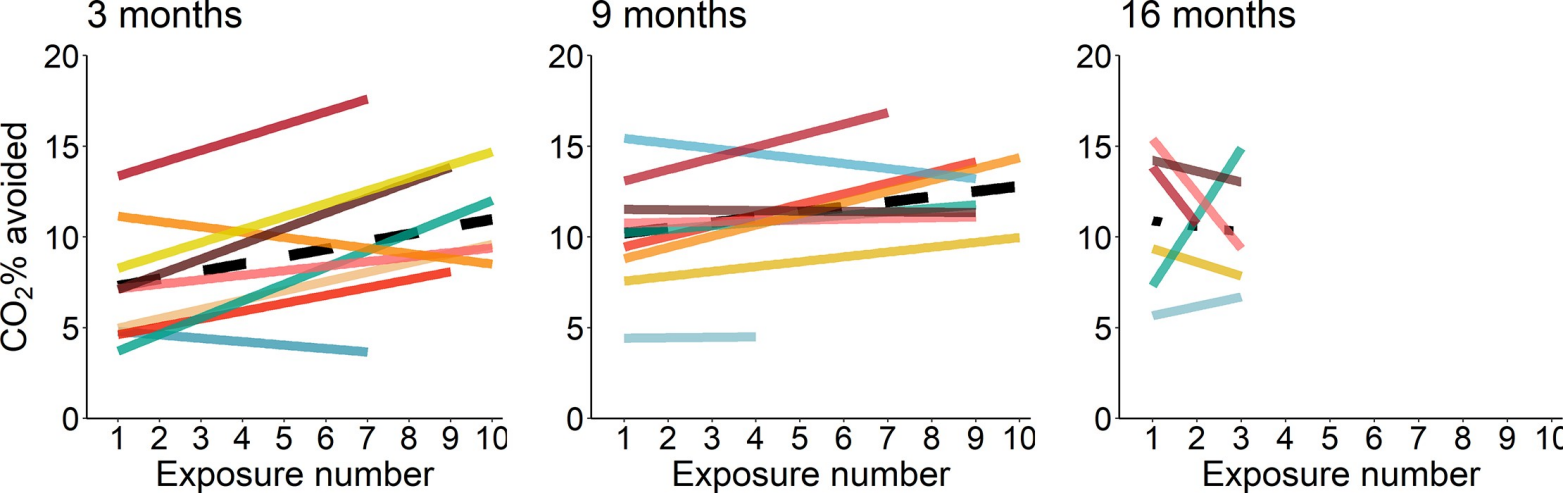
Effect of repeated exposures in aversion to CO_2_ at 3, 9 and 16 months of age. Individuals are represented by solid lines; the color identifying individuals are consistent across ages. Dashed lines represent the change in CO_2_ tolerance with repeated exposures within each age.

**Fig 4:**
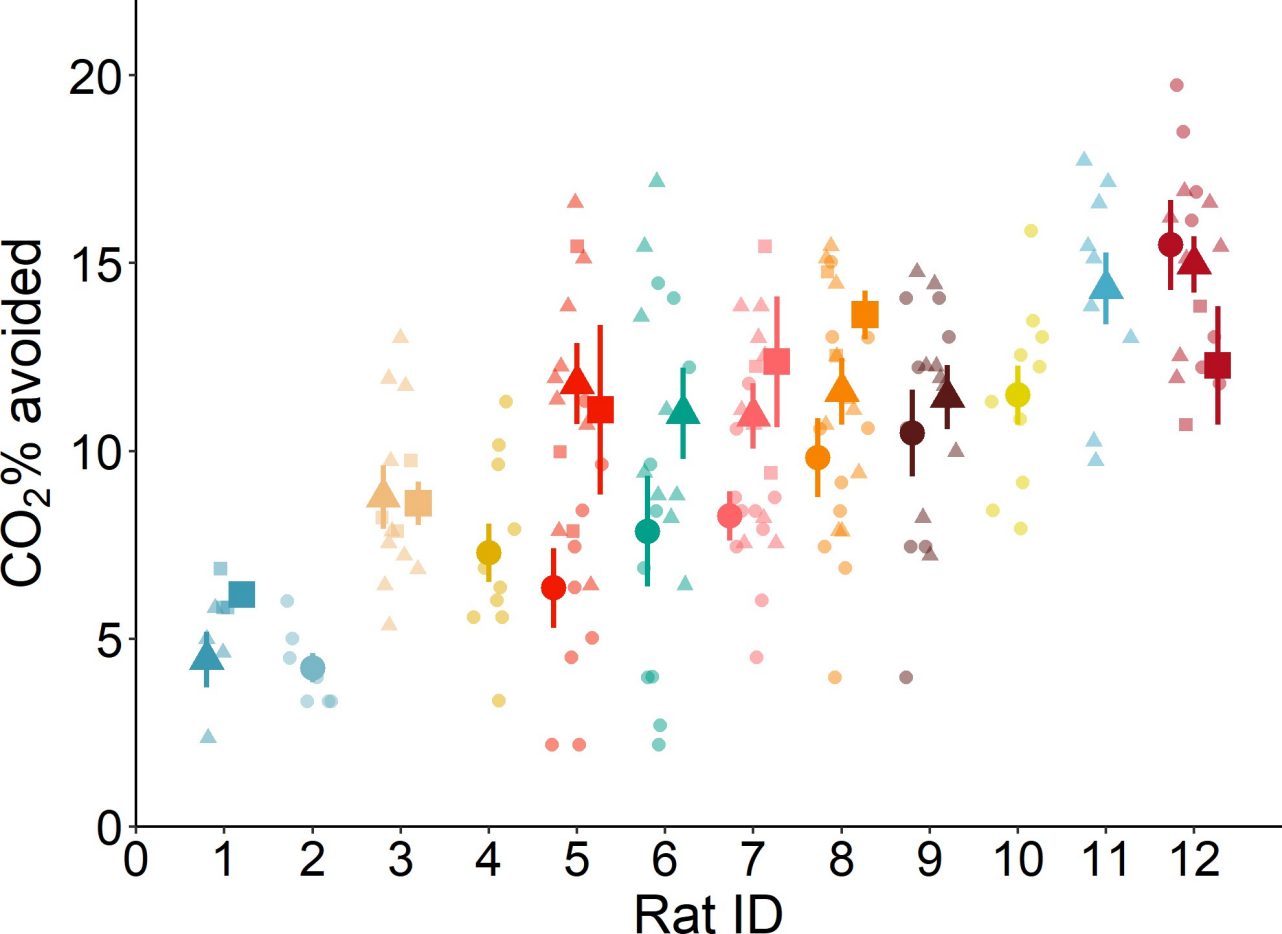
Individual differences in aversion to CO_2_, arranged from the least to the most tolerant rat. Bigger and solid shapes indicate the mean and error bars show the standard error for each individual rat within each age. Smaller and transparent shapes represent observation from each rat at each age. Each age is presented with a different shape (circles: 3 months, triangles: 9 months, squares: 16 months) and each color represents an individual rat.

During control trials, rats left the cage after 4.8 ± 1.8 min and ate 18 ± 3 Cheerios. In all test trials rats avoided CO_2_ before recumbency. Concentrations of CO_2_ avoided by rats increased with repeated exposures (F_1,148_ = 17.83, p < 0.0001; n = 12; Fig 3). Aversion to CO_2_ was affected by age (F_2,10_ = 6.09, p < 0.02; n = 12). Compared to 3 months of age (8.4 ± 0.9% CO_2_), rats tolerated higher CO_2_ concentrations at 9 months of age (p = 0.02; 10.9 ± 0.9% CO_2_). We found no evidence for an interaction between age and exposure number (F_2,148_ = 0.57, p = 0.57; n = 12). Rat identity and series identity within rat explained 45% (Likelihood ratio test: LR = 61.67, p < 0.001; n = 12) and 5% (LR = 1.45, p = 0.21; n = 12) of the variation in the CO_2_ concentrations avoided, respectively. CO_2_% avoided was repeatable (rat identity adjusted repeatability: R = 0.46, p < 0.01; series identity: R = 0.07, p = 0.06; n = 12; Fig 4); with average CO_2_ concentrations avoided ranging between 4 and 15% among rats.

### Experiment 2: Sweet reward motivation

**Fig 5:**
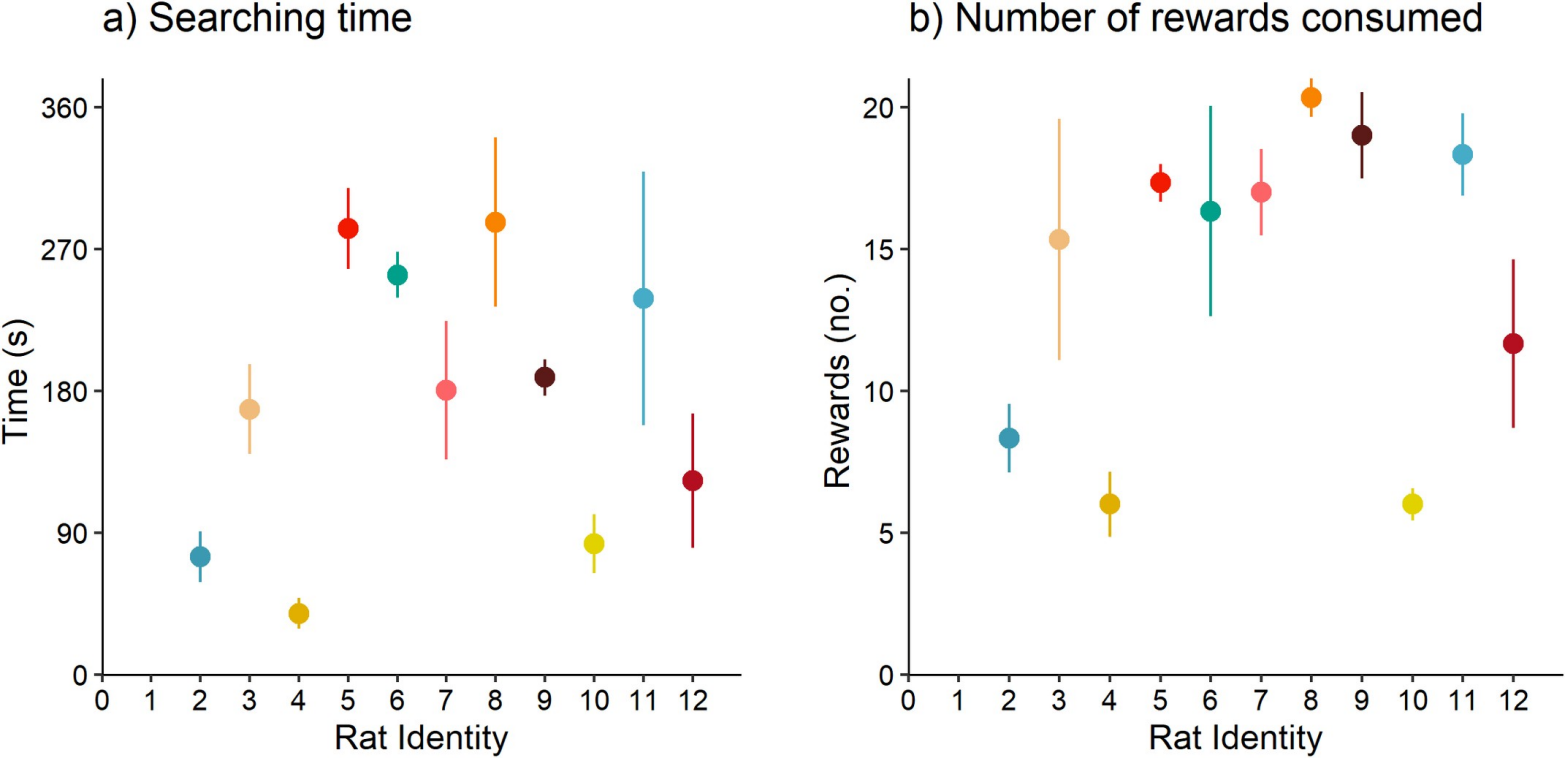
Individual differences in sweet reward motivation. Individual rat (n = 11) mean (± SE) a) searching time, and b) rewards consumed, across three sweet reward motivation trials. Rat identity follows that shown in Fig 4.

**Fig 6:**
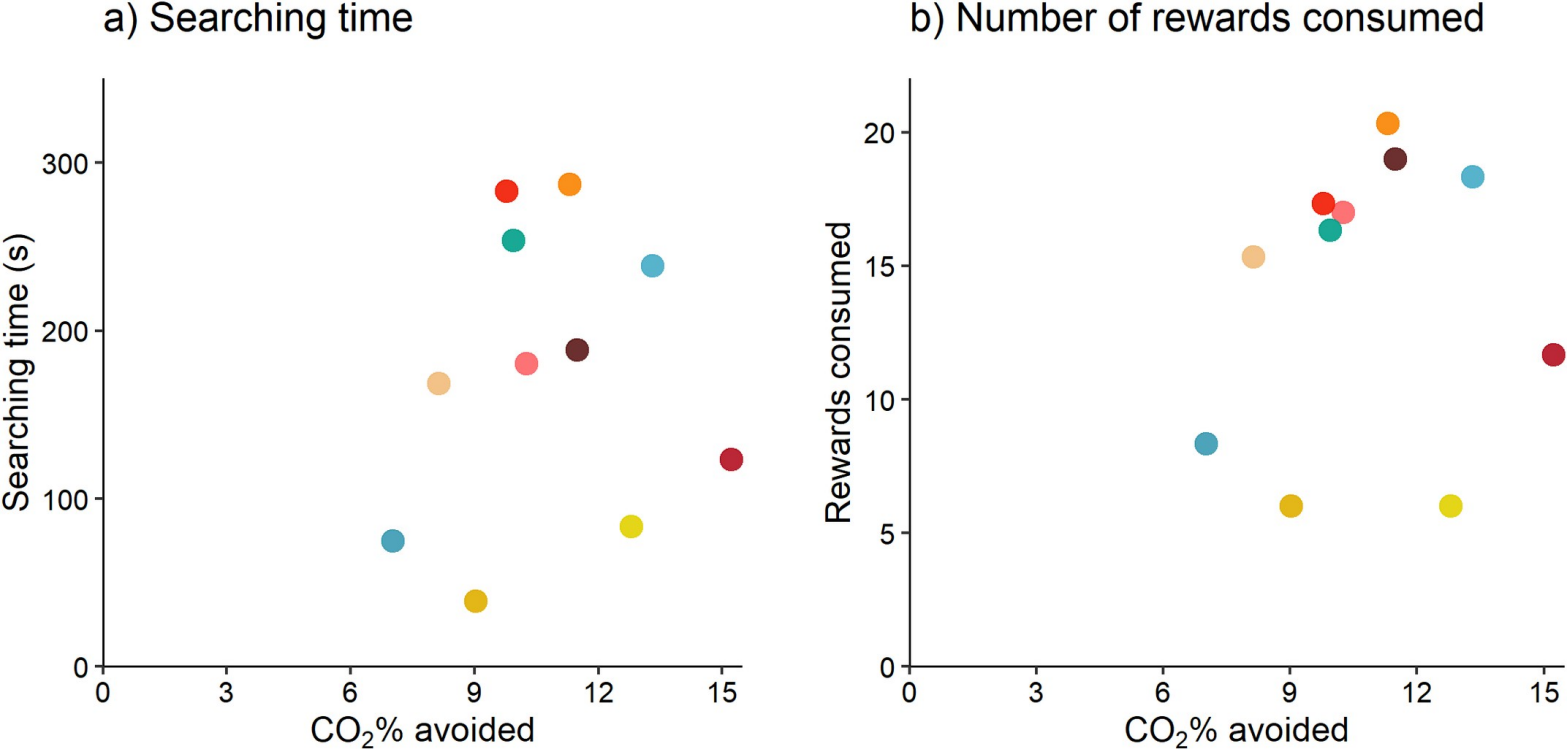
Relationship between aversion to CO_2_ and sweet reward motivation. Each dot represents an individual rat’s point estimate (obtained from the BLUPs of the random effects of 1000 simulations) of aversion to CO_2_ and their average a) searching time, and b) rewards consumed, from the sweet reward motivation trials (n = 11).

Trial number did not affect rewards consumed or total searching time (sweet rewards consumed: F_2,20_ = 0.33, p = 0.72; searching time: F_2,20_ = 0.19, p = 0.83; n = 11; Fig 5). Rats spent on average 174 ± 17.2 s searching for rewards, and consumed on average 14.2 ± 1.04 sweet rewards. The random intercept (rat identity) explained 56% (LRT = 9.9, p < 0.01, n = 11) and 60% (LRT = 11.61, p < 0.001, n = 11) of the variation in searching time and rewards consumed, respectively. Searching time and sweet rewards consumed were repeatable (R = 0.56, p < 0.0001, and R = 0.60, p < 0.001, respectively; n = 11). However, aversion to CO_2_ was not related to searching time (Pearson correlation test: r = 0.13, p = 0.71; n = 11) or rewards consumed (r = 0.13, p = 0.71; n = 11) in sweet reward motivation trials (Fig 6).

### Experiment 3: Regulatory focus

**Fig 7:**
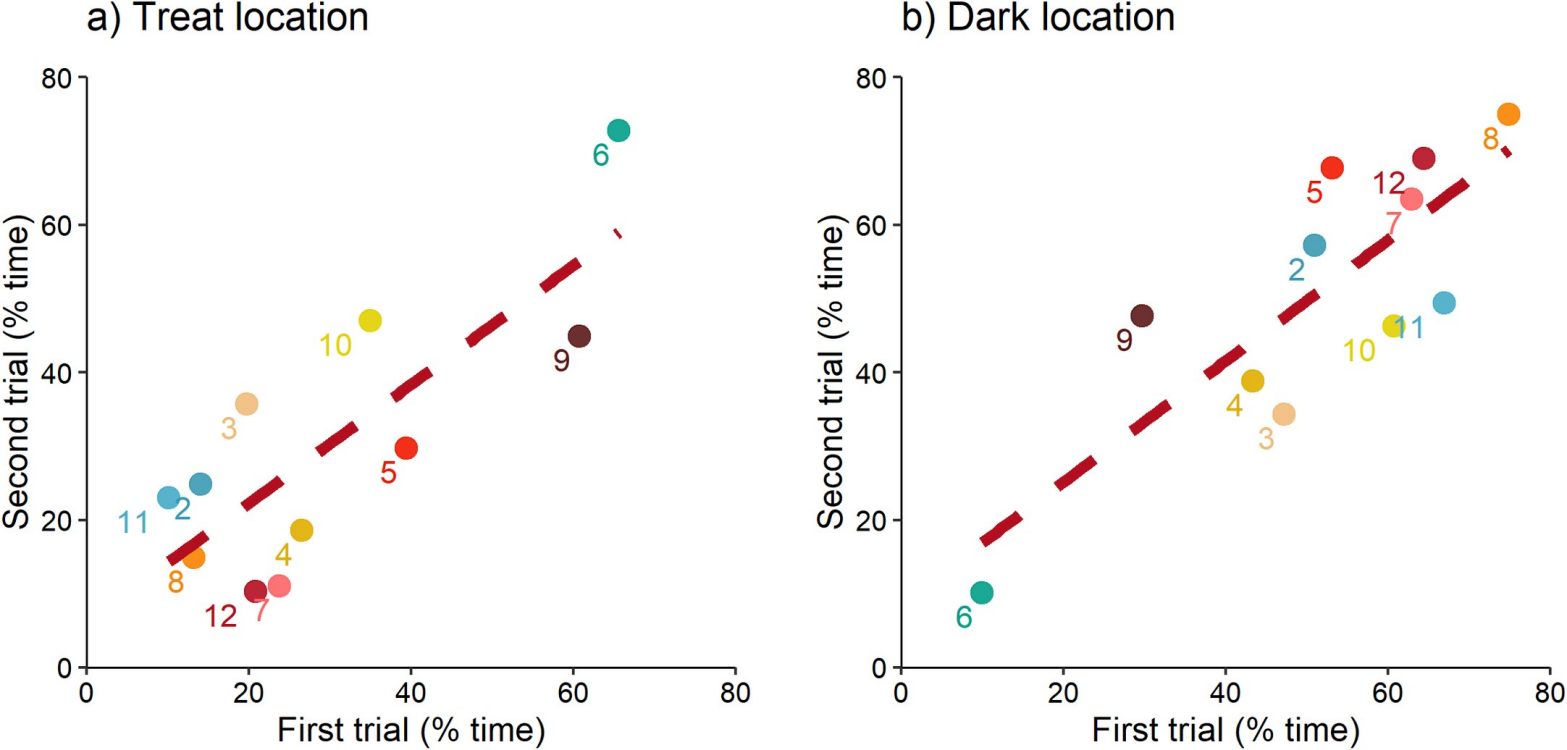
Regulatory focus consistency for individual rats (n = 11) tested in two trials; results are shown separately for measures of a) treat location time (promotion motivation), and b) dark location time (prevention motivation). Rat identity is given by a number following the order presented in Fig 4.

Rats spent on average 31% and 51% of the test time in the treat and dark locations, respectively. Across the two test trials, rats consistently varied in the percentage of time spent in these locations (treat: r = 0.80, p < 0.01; dark: r = 0.81, p < 0.01; n = 11 rats; Fig 7). Aversion to CO_2_ was not related to the percentage of time spent in the treat (r = 0.34, p = 0.3; n = 11) or dark locations (r = -0.09, p = 0.79; n = 11).

## Discussion

In agreement with previous studies [15, 24], we found that rat aversion to CO_2_ consistently varied among rats, ranging between 4 and 15% CO_2_. As frequently occurs in longitudinal studies, despite the effort to obtain measurements of aversion to CO_2_ from all subjects at all time points, data from some rats were missing for some ages. Despite this unbalance data, our method of analysis had sufficient power test variation among individuals. To our knowledge, the current study is the first to show that rat individual thresholds of aversion to CO_2_ are stable and highly repeatable across repeated exposures and across different ages (R = 0.46; the average repeatability estimates across behaviours and among taxa has been shown to be 0.37 [30]).

Previous studies have reported high between-individual variation in rat responses to CO_2_ during forced exposure [7, 9, 14], choice [31, 32], and aversion tests [10, 11, 33], but the source of this variation remained unexplained. Rat defence behaviours are plastic, varying with environmental familiarity (i.e. habituation) [34–36], situational contingencies (for example, threat proximity and possibility to escape) [37–39], and with specific conditions prior to or during testing [40–45]. Although we found that the thresholds of aversion to CO_2_ were higher at older ages, at 3 months of age rats were exposed to a higher flowrate than that used at later ages, impeding our ability to draw age-related inferences. Contrary to the general trend, as it can be seen in Fig 4, rat number 12 experienced a decrease in aversion to CO_2_ measured at ages 9 and 16 months when compared to age 3 months. This could indicate the presence of individual differences in plasticity; however, the current study lacks power to make strong claims about rat differences in slopes. Consistent with previous results from our research group [9], we found that thresholds of aversion to CO_2_ increased over repeated exposures at 3 and 9 months of age. This result is also consistent with human studies showing that chemoreceptor sensitivity [46] and feelings of anxiety [47, 48] decrease with habituation to CO_2_ inhalation. Overall, these results indicate that aversion to CO_2_ is plastic and sensitive to habituation.

Behaviours consistent across time and contexts are often referred to as personality traits. These individual differences are more or less permanent characteristics that distinguish individuals from one another [49, 50]. For example, within the same strain, the degree to which rats explore novel environments is consistent between four and eight months of age [51]. Individual differences in this behavioural trait are heritable; two lines, originating from rats that differed in active avoidance acquisition (i.e. Roman high avoidance and Roman low avoidance), consistently differ in their degree of exploration of novel environments [52]. Our results showed that rat thresholds of aversion to CO_2_ vary among individuals, are highly repeatable, and can be considered a lasting characteristic of the individual (i.e. personality trait).

In the current study we tested individual differences in motivation to access a sweet reward using a modified approach-avoidance apparatus. Traditionally, rat motivation is measured through a progressive ratio schedule, in which the cost of gaining a reward is increased. Animals continue to invest as the required effort increases, until the cost is higher than the value of the reward [53]. In the current study, rat motivation was assessed in an experimental setting similar to that used to assess differences in foraging wild rodents [54, 55]. The individual’s motivation to engage in a goal directed behaviour, like searching for food, is affected by expectancy and value [56, 57]. Motivation is expected to be higher as the likelihood of success and the value of the resources increase. When foraging in a patch, animals experience a decrease in the rate at which resources are found as they consume the available items (diminishing returns); this decrease imposes a trade-off between investing time searching for resources and leaving the patch (marginal value theorem) [58]. In the current experiment, rats searched for access to sweet rewards that were hidden in a layer of sand, and rewards were progressively dispersed across trials. The assumption was that rats would invest time searching and digging to gain access to the rewards until the required effort surpassed the value of the reward.

We found that rat motivation for sweet rewards varied among individuals. For example, one rat invested on average 5 min searching and consumed all rewards, and another rat consumed on average just 6 rewards and spent less than 40 s searching. We also found that motivation for sweet rewards was highly repeatable and not affected by repeated testing. These results align with what has been reported in the literature; rat preference and motivation for sucrose is a stable personality trait [19, 59] that does not change with repeated testing [19]. High sucrose consumers ingest more than double the intake of low consumers [18, 60, 61], but among-individual variation in sucrose preference is not related to variation in food consumption [60]. Under a progressive ratio schedule, high consumers work harder than low consumers to earn sucrose [17].

We found no evidence that individual differences in motivation for sweet rewards were related to aversion to CO_2_. However, it is important to note that rats that showed low motivation for sweet rewards frequently failed to meet the training criterion for the approach-avoidance test. This result suggests that a bias of approach-avoidance tests is that only reward-motivated rats are likely to be selected.

In the current study rats spent a similar amount of time in the treat location (31% of the test time) but less time in the dark location (51% of the test time), compared to results from Franks and colleagues [23]. It is likely that the lower time in the dark location was due to methodological differences. Franks and colleagues kept the light off for 30 s, or while the subject stayed in the dark location, whichever was longer. In our study the light was turned on after 30 s or when the rat left the dark location, whichever occurred first. We argue that our experimental methodology allows for the assessment of prevention and promotion foci since rats frequently brought food rewards to consume in the dark location, and rats consistently varied in their motivation to approach gains (promotion motivation) and pursue darkness (prevention motivation). These results correspond to those previously reported. For example, rats that consistently pursued darkness in the modified open field, also consistently spent more time burying a noxious object [23]. High prevention motivated rats avoided risk, but also approach potential threats to maintain safety. In the current study, we found no evidence for a relationship between individual differences in the strength of promotion or prevention motivation and rat aversion to CO_2_, indicating that personality differences in regulatory focus are not related to aversion to CO_2_ in approach-avoidance.

Variation among human subjects in the felt experience (i.e. conscious awareness of emotions) during CO_2_ inhalation is well documented. The increase in feelings of anxiety is eight times higher in individuals that are more responsive to CO_2_, than that of less responsive individuals [62]. Feelings of anxiety and experiences of panic due to ~ 7% CO_2_ inhalation are consistent between repeated inhalations [62, 63]. Vulnerability to CO_2_-induced anxiety and panic increases in people diagnosed with panic disorder [3, 64] and individuals with a first-degree relative diagnosed with panic disorder [4, 65, 66]. Thus, human CO_2_ sensitivity involves stable individual differences in the emotional response to CO_2_. We found that individual differences in rat thresholds of aversion to CO_2_ were stable and consistent, and not related to sweet reward motivation or the strength of promotion and prevention motivations. It is likely that individual differences in the affective states experienced are the underlying cause of among-rat variation in aversion to CO_2_ (i.e. CO_2_ sensitivity), indicating that some rats experience a more aversive emotional response when exposed to CO_2_.

## Conclusion

Variation in rat aversion to CO_2_ was repeatable through multiple exposures and across three different ages but was not related to individual differences in motivation for sweet rewards, promotion or prevention foci. These results indicate that individual differences in aversion to CO_2_ reflects variation in CO_2_ sensitivity.

## Supporting information

S1 File (DOCX)

S1 Data (XLSX)
