# Supplementary material for: Individual differences in rat sensitivity to CO2
Source: PLoS One. 2021 Jan 22;16(1):e0245347. doi: 10.1371/journal.pone.0245347 (PMC7822239; doi:10.1371/journal.pone.0245347)
Supplement: S1 File — (DOCX) [file pone.0245347.s001.docx]

**Individual differences in rat sensitivity to CO_2_**

Lucía Améndola, Anna Ratuski, Daniel. M. Weary

**Supporting Methods S1: Alternative analysis**

**Experiment 1: aversion to CO_2_**

We estimated the CO_2_% concentrations at the time when rats exited the bottom cage (i.e. CO_2_% avoided), using the average concentration of CO_2_ at each time point (measured every 0.2 s) during the 12 CO_2_ flow trials. We used three linear mixed models (“nlme” R-package), one per each age (3, 9 and 16 months of age) with the response variable CO_2_% avoided in approach-avoidance tests. The models included exposure number as a covariate, and rat identity as the random intercept. Exposure number was mean centered and standardized to 2 standard deviations. The significance of the random intercepts was evaluated using the likelihood ratio test (LRT). We estimated repeatability (R; “rptR” R-package) of CO_2_% avoided adjusted at each age adjusting for exposure number (adjusted repeatability for Gaussian data).

For rats with similar experiences (i.e. tested with CO_2_ at both 3 and 9 months of age; n = 6 rats), from the analysis at 9 moths of age, we obtained the BLUPs of the random effects from 1000 simulations (“arm” R-package). We used the average number of sweet rewards consumed and total searching time across trials from the six rats with similar experiences. The relationship between the two measures of rat motivation for sweet rewards and the average BLUPs of CO_2_% avoided in approach-avoidance tests was assessed using Spearman rank correlation tests. For each of the six rats with similar experiences, we estimated the average percentage of test time spent in the treat (and dark) location across the two trials. Again, we used Spearman rank correlation tests to assess the relationship between promotion (and prevention) focus and the average BLUPs of CO_2_% avoided.

**Results**

**Experiment 1: aversion to CO_2_**

At 3 and 9 months of age, concentrations of CO_2_ avoided by rats increased with repeated exposures. Rat identity explained the variation in the CO_2_ concentrations avoided at all ages and CO_2_% avoided was repeatable within each age (Table 1).

Table 1. Analysis of the CO_2_ concentrations avoided at 3, 9 and 16 months of age.

|  |  | Exposure number | | | | Random intercept | | | Repeatability | |
| --- | --- | --- | --- | --- | --- | --- | --- | --- | --- | --- |
|  | LSM ± se | β | df | F | p | % var. | LRT | p | R | p |
| Age  (mo.) |  |  |  |  |  |  |  |  |  |  |
| 3 | 9.22 ± 1 | 2.56 | 1,72 | 14.72 | **<0.001** | 52% | 32.65 | **<0.001** | 0.55 | **<0.001** |
| 9 | 11.3 ± 0.88 | 1.51 | 1,66 | 4.65 | **<0.05** | 44.5% | 18.11 | **<0.001** | 0.48 | **<0.001** |
| 16 | 9.90 ± 2.73 | -1.23 | 1,10 | 0.08 | 0.78 | 47% | 3.5 | 0.06 | 0.5 | **<0.05** |

LSM: lest square means; se: standard error; β: slope; df: degrees of freedom; LRT: Likelihood ratio test

Aversion to CO_2_ was not related to searching time (Pearson correlation test: rho = -0.14, p = 0.8; n = 6) or rewards consumed (rho = -0.14, p = 0.8; n = 6) in sweet reward motivation trials. Aversion to CO_2_ was also not related to the percentage of time spent in the treat (rho =-0.2, p = 0.71; n = 6) or dark locations (rho = 0.2, p = 0.71; n = 6) in the regulatory focus trials.

**Supporting Methods S2: Aversion to CO_2_ random regression models**

For experiment 1, we were interested in assessing between-individual variation in aversion to CO_2_ through repeated exposures and across different ages (3, 9 and 16 months of age). When individual responses are obtained repeatedly across an environmental gradient, it is possible to estimate between-individual variation in the average response (i.e. personality) and between-individual variation in within-individual changes over the environmental gradient (i.e. individual differences in behavioural plasticity). Using behavioural reaction norms, individuals can be characterized by a combination of an intercept (between-individual variation in the average response) and a slope (behavioral plasticity)^1^. In experiment 1, repeated measures of aversion to CO_2_ were taken on a shorter (repeated exposures within age) and a longer temporal scale (across 3, 9 and 16 months of age)^2^. To assess reaction norm components, individuals were grouped by rat identity (rats 1 to 12), and a unique combination of the individual rat by the age at which observations were taken (i.e. series). We used Random Regression Models with the response variable CO_2_% avoided, age (mean centered and standardized to 2 standard deviations) as a fixed factor, exposure number within age (mean centered and standardized to 2 standard deviations) as covariate and its interaction. The full model included series identity within rat identity as random intercept, and exposure number as a random slope. We compared this model to a model that excluded exposure number as random slope (Table 1).

Table 1. Comparison between models including (co)variance components of the random effects.

|  | Full model |  |  |  | Model 2 |  |  |
| --- | --- | --- | --- | --- | --- | --- | --- |
|  |  | δ^2^ | δ |  | δ^2^ | δ |  |
| **Random effects** |  |  |  |  |  |  |  |
| *Between-individual rat* | Intercept | 7.15 |  |  | 6.82 |  |  |
|  | Exposure number slope | 0.02 |  |  | - |  |  |
|  | Covariance |  | 0.42 |  |  | - |  |
|  |  |  |  |  |  |  |  |
| *Between-series within rat* | Intercept | 0.85 |  |  | 0.78 |  |  |
|  | Exposure number slope | 0.60 |  |  | - |  |  |
|  | Covariance |  | 0.23 |  |  | - |  |
|  |  |  |  |  |  |  |  |
|  | Residual | 7.30 |  |  | 7.45 |  |  |
|  |  |  |  |  |  |  |  |
|  | Deviance | 885.54 |  |  | 885.84 |  |  |
|  | χ^2^ | - |  |  | 0.30 |  |  |
|  | p | - |  |  | 0.99 |  |  |
|  |  |  |  |  |  |  |  |

**References**

1. Dingemanse, N. J., Kazem, A. J. N., Réale, D. & Wright, J. Behavioural reaction norms: animal personality meets individual plasticity. *Trends Ecol. Evol.* **25,** 81-89 (2010). doi:10.1016/j.tree.2009.07.013.
2. Araya‐Ajoy, Y.G., Mathot, K.J. and Dingemanse, N.J. An approach to estimate short‐term, long‐term and reaction norm repeatability. *Methods Ecol Evol.* **6**, 1462-1473 (2015).

**Supporting Methods S3:** **Power of analysis for random effects**

We assessed the power to detect significant random intercepts and slopes when including rat identity and series identity as random intercepts and exposure number as random slope using the function EAMM of the R-package “pamm”^1^. The average power to detect the random intercept and slope were 0.87 and 0.67 respectively (Fig 1).


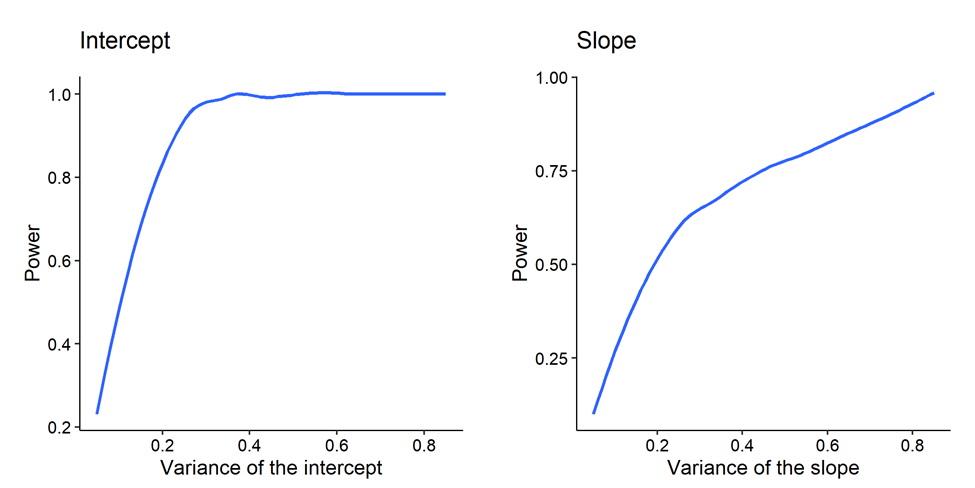


Fig 1. Power to detect random intercepts (left) and slopes (right) for a Random Regression Model that included age and exposure number (both mean centered and standardized to 2 deviations) as fixed effects, series identity within rat identity as random intercept and exposure number as random slope.

**References**

1. Martin, J.G., Nussey, D.H., Wilson, A.J. and Réale, D. Measuring individual differences in reaction norms in field and experimental studies: a power analysis of random regression models. *Methods Ecol Evol.* **2**, 362-374 (2011).

**Supporting Methods S4: Model diagnostics**

We analyzed the response variable CO_2_% avoided in approach-avoidance tests, with a linear mixed model that included age as a fixed factor, exposure number (within age) as a covariate, the interaction between age and exposure number, and series identity within rat identity as a random intercept. We compared the fit of this model to models with different variance-covariance structures (i.e. autoregressive and autoregressive with heterogeneous variances). The homoscedastic model with compound symmetrical correlation structure had the best fit (i.e. the lowest Akaike’s Information Criterion values; data not reported). Normality of the within-group errors (Figs 1 and 2) and the random effects (Fig 3), and constant variance (Fig 4) were visually assessed.


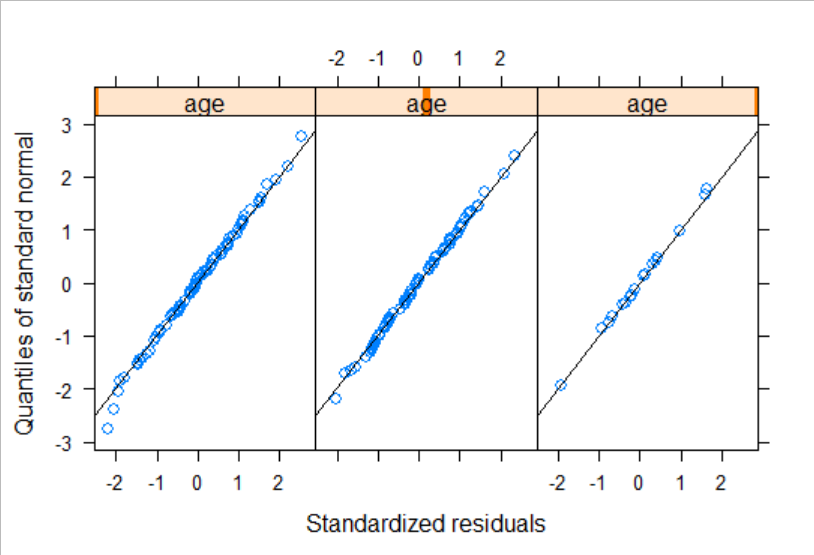


Fig 1. Normal plot of residuals for the model considering within age errors.


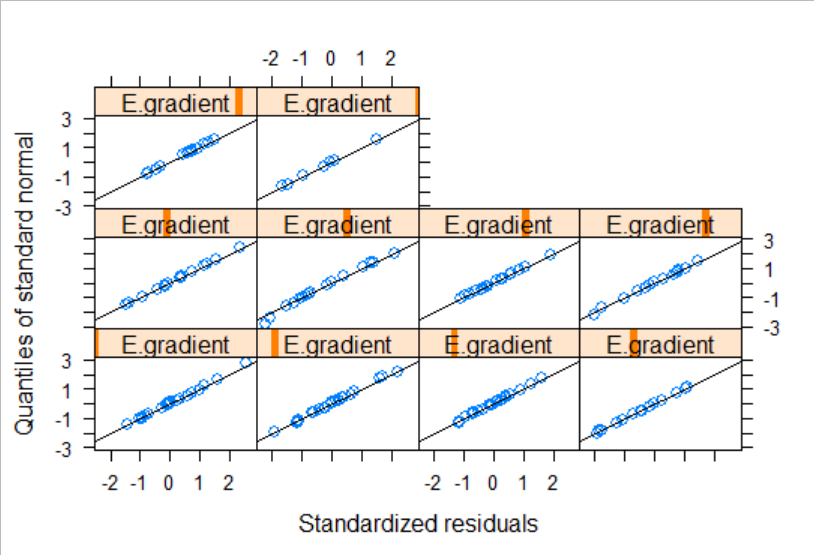


Fig 2. Normal plot of residuals for the model considering within exposure number errors.


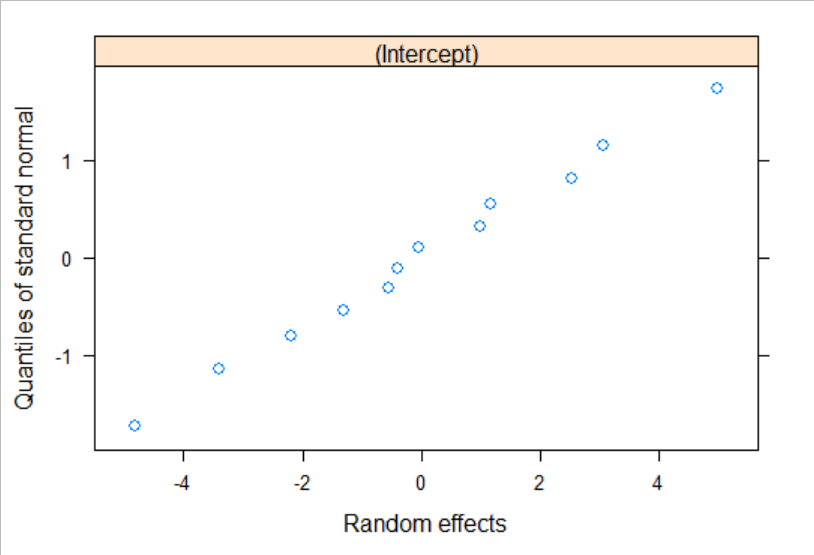


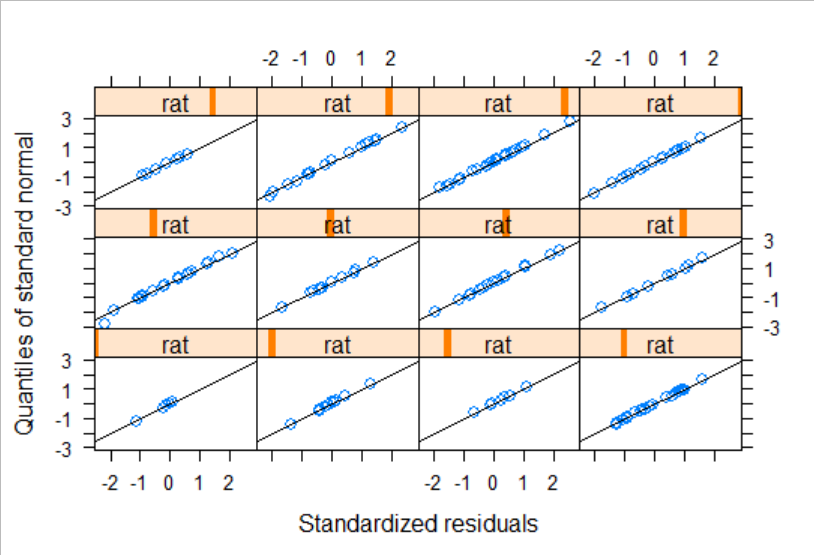

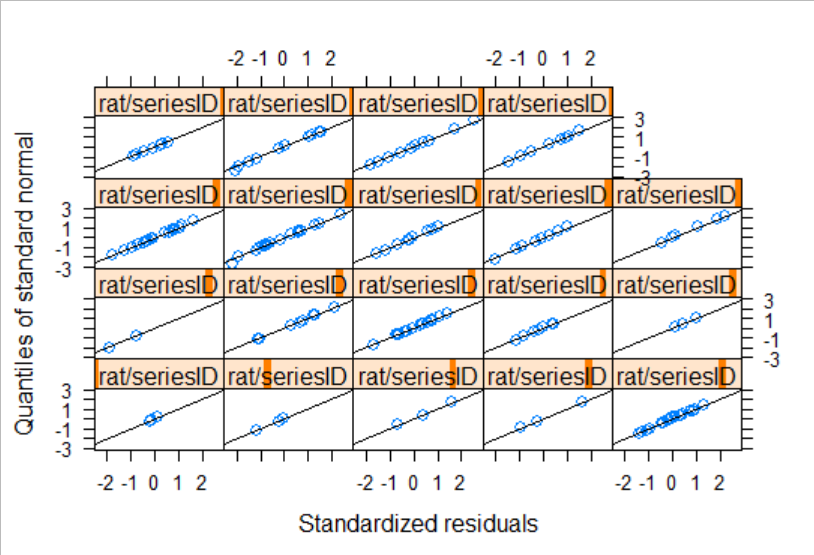


Fig 3. Normal plots of random effects.


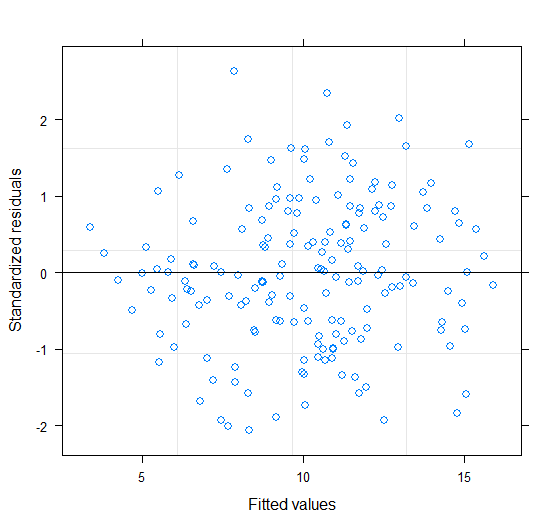


Fig 4. Constant variance. Standardize residuals versus fitted values.
